# The release of adhesions improves outcome following minimally invasive repair of Achilles tendon rupture

**DOI:** 10.1007/s00167-021-06767-6

**Published:** 2021-10-18

**Authors:** Michael R. Carmont, Sara Brandt Knutsson, Annelie Brorsson, Jón Karlsson, Katarina Nilsson-Helander

**Affiliations:** 1 The Department of Trauma and Orthopaedic Surgery, Princess Royal Hospital, Shrewsbury and Telford Hospital NHS Trust, Shropshire, UK; 2 The Department of Orthopaedics, Institute of Clinical Sciences at Sahlgrenska Academy, Gothenburg University, Gothenburg, Sweden

**Keywords:** Achilles tendon repair, Adhesions, Outcome

## Abstract

**Purpose:**

Operative repair of Achilles tendon rupture may lead to complications, which influence outcome adversely. The aim of this study was to determine the incidence, impact and response to treatment of post-operative adhesions.

**Methods:**

From February 2009 to 2021, 248 patients operated on with percutaneous or minimally invasive surgical repair have been prospectively evaluated using the Achilles tendon Total Rupture Score (ATRS) and Heel-Rise Height Index (HRHI), following acute Achilles tendon rupture.

**Results:**

Fourteen (5.6%) patients were identified as having adhesions. Four patients reported superficial adhesions and ten patients reported a deeper tightness of the tendon. At a mean (SD) of 10.5 (2.3) months following repair, the overall ATRS was at a median (IQR) 65 (44.5–78) points and (HRHI) was mean (SD) 81.5 (13.5)%. Of those deemed to have deep adhesions the antero-posterior diameter of the tendon was at mean (SD) 15.6 (4.6) mm. Open release of superficial adhesions resulted in improved ATRS in all patients. Endoscopic debridement anterior to the Achilles tendon led to alleviation of symptoms of tightness and discomfort from deep adhesions and improved outcome in terms of the ATRS score. At a mean (SD) of 15.9 (3.3)-month follow-up from initial rupture and repair, the patients reported at median (IQR) ATRS scores of 85 (64.8–92.8) points, Tegner level 5 (3–9) and mean (SD) HRHI 86.2 (9.5)%. Patients significantly improved both ATRS and HRHI following release at median (IQR) of 16.5 (− 1.8–29.3) points (*p* = 0.041) and mean (SD) 5.6 (8.3)% (*p* = 0.043).

**Conclusions:**

The incidence of patient-reported adhesions following minimally invasive repair of Achilles tendon rupture was estimated to be 5.6%. The occurrence of superficial adhesions was associated with a lower outcome scores as well as symptoms of anterior tendon tightness and stiffness were associated with a lower score in most patients. Surgical release of adhesions led to a significant improvement in outcome.

## Introduction

Meta-analyses of the management following Achilles tendon rupture consistently report lower rates of re-rupture and higher rates of complications with surgical than non-surgical treatment [30, 32, 34]. Randomised controlled trials, however, have also shown greater plantar flexion strength [19, 30, 35, 37] and performance in functional and sports-related tasks [31], with less elongation of the tendon [14] following surgical repair. Minimally invasive or percutaneous surgery leads to good outcome with reduced wound complication rates, although wound problems may still occur [12].

Superficial adhesions, such as skin tethering and deep adhesions causing tendon pain, have repeatedly been reported as complications of rupture for both surgical and non-surgical management with rates of 5–7.1% [21, 23–25, 28, 29]. Carmont et al. [7] reported that complications, such as superficial wound infection, cast-related wounds, skin complications and sural nerve injury have not been shown to influence patient-reported outcome scores at 12 months following repair. Metz et al. [22], however, found that when minor wound complications, including adhesions were grouped together, patients reported significantly lower Achilles tendon Total Rupture Scores (ATRS) with a rate of 6% of patients having a score of 80 points, compared with 89 points for those without such complications (*p* = 0.0445) at 6.2-year follow-up. The impact of adhesions, specifically on early clinical outcome and the effect of specific intervention for adhesions has, however, not yet been reported. The presence of adhesions may reduce the overall outcome of patients receiving operative treatment. The subsequent operative treatment of adhesions may improve outcome following Achilles tendon rupture.

The aim of this study was to determine the incidence of adhesions, the impact of adhesions on patient-reported outcome and function following Achilles tendon repair together with the effect of surgical adhesiolysis on outcome.

## Materials and methods

The Research and Innovation Department of NHS Trust deemed this study to be service evaluation and formal ethical approval was, therefore, waived.

From February 2009 to March 2021, 248 patients received operative repairs for Achilles tendon ruptures by the same surgeon and received the same physiotherapy instructions. Repairs were performed using percutaneous [7] or minimally invasive [8, 9] techniques followed by immediate loading on the metatarsal heads, with patients mobilising in a protective synthetic cast in full plantar flexion [7–9]. Repairs were performed with either absorbable monofilament or non-absorbable braided sutures. A small number of heavy patients (> 110 kg) or those with a short distal stump were deemed to be high risk of re-rupture and so the distal pass of the non-absorbable suture passage was placed through a transosseous calcaneal tunnel [4]. The main surgical incision at the repair site was typically 2 cm long. One hundred and three patients received repairs using absorbable monofilament sutures and 145 patients, non-absorbable braided sutures. Seventeen patients received non-absorbable sutures passed distally through the transosseous calcaneal tunnel.

At 2 weeks following repair patients were encouraged to undertake plantar flexion, inversion and eversion exercises, however, all mobilisation was protected by wearing an anterior shell only and using elbow crutches. Walking was permitted using a 1.5 cm heel wedge at the 6-week time point and physiotherapy commenced consisting of calf strengthening exercises, although plyometric and active stretching exercises were only permitted after 3 months.

### Evaluation

**Fig. 1:**
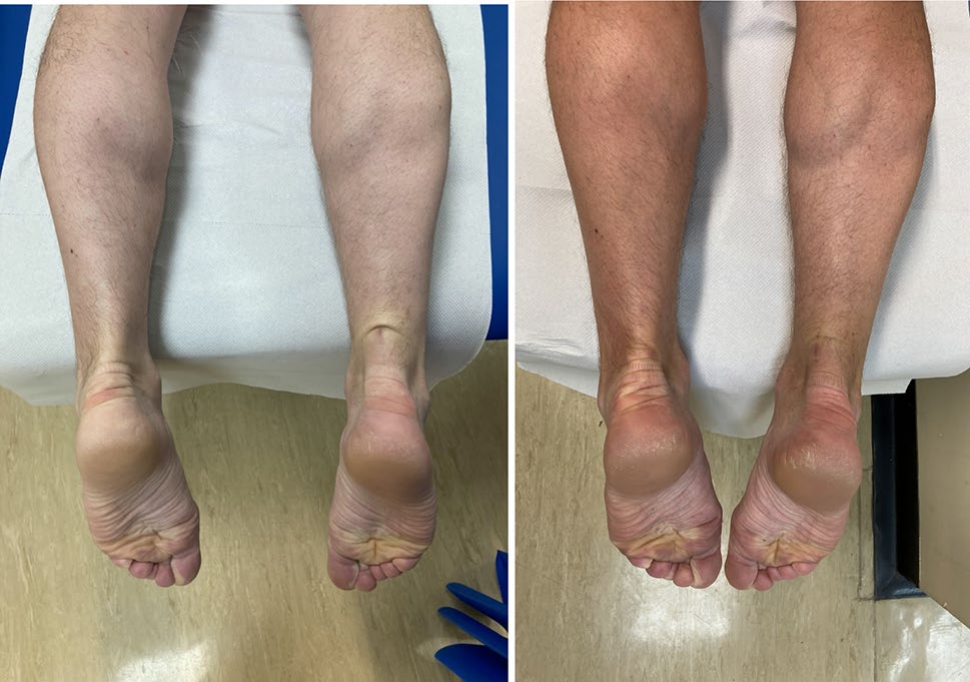
Superficial adhesion causing symptoms of superficial stiffness and tethering at 12 months following repair (left) and then at 3 months following release of adhesion (right)

Patients were evaluated during routine follow-up at 3, 6, 9 and 12 month following repair. Evaluation consisted of noting the presence of any symptoms, limitations including the Achilles tendon Total Rupture Score (ATRS) [27], the patient’s current Tegner score [36], the Achilles Tendon Resting Angle (ATRA) [8, 9], the heel-rise height index (HRHI) [13]. The presence of atypical symptoms and signs were documented, particularly symptoms of pulling, tightness, stiffness and any pain be it either superficial or deep to the Achilles tendon. The skin was inspected during range of movement for the visible tethering of superficial adhesions and a lack of “bounce” or compliance during movement of the tendon and heel-rise exercises (Fig. 1). Superficial adhesions were determined by the presence of subcutaneous tethering associated with pulling, tightness and stiffness. Deep adhesions were associated with a pain deeper to the Achilles tendon with tightness and stiffness and were usually indicated with the palm of the hand around the heel. The tendon was noted not to bounce, stretch and recoil, as much as on the non-injured side.

### Adhesiolysis interventions

Subcutaneous adhesiolysis was performed without tourniquet, using 2% chlorhexidine skin preparation and local anaesthetic field block of 10 ml 0.5% Bupivacaine with 1:200,000 Adrenaline. A 2.5-cm longitudinal incision was made medial to the skin adhesion with deep dissection to the fascia cruris. By asking the patient to carefully plantar flex and dorsiflex the ankle, the adhesion was identified. Tough adhesive scar tissue was released using a scalpel. Subcutaneous polyglactin sutures were used with subcuticular nylon for the skin.

**Fig. 2:**
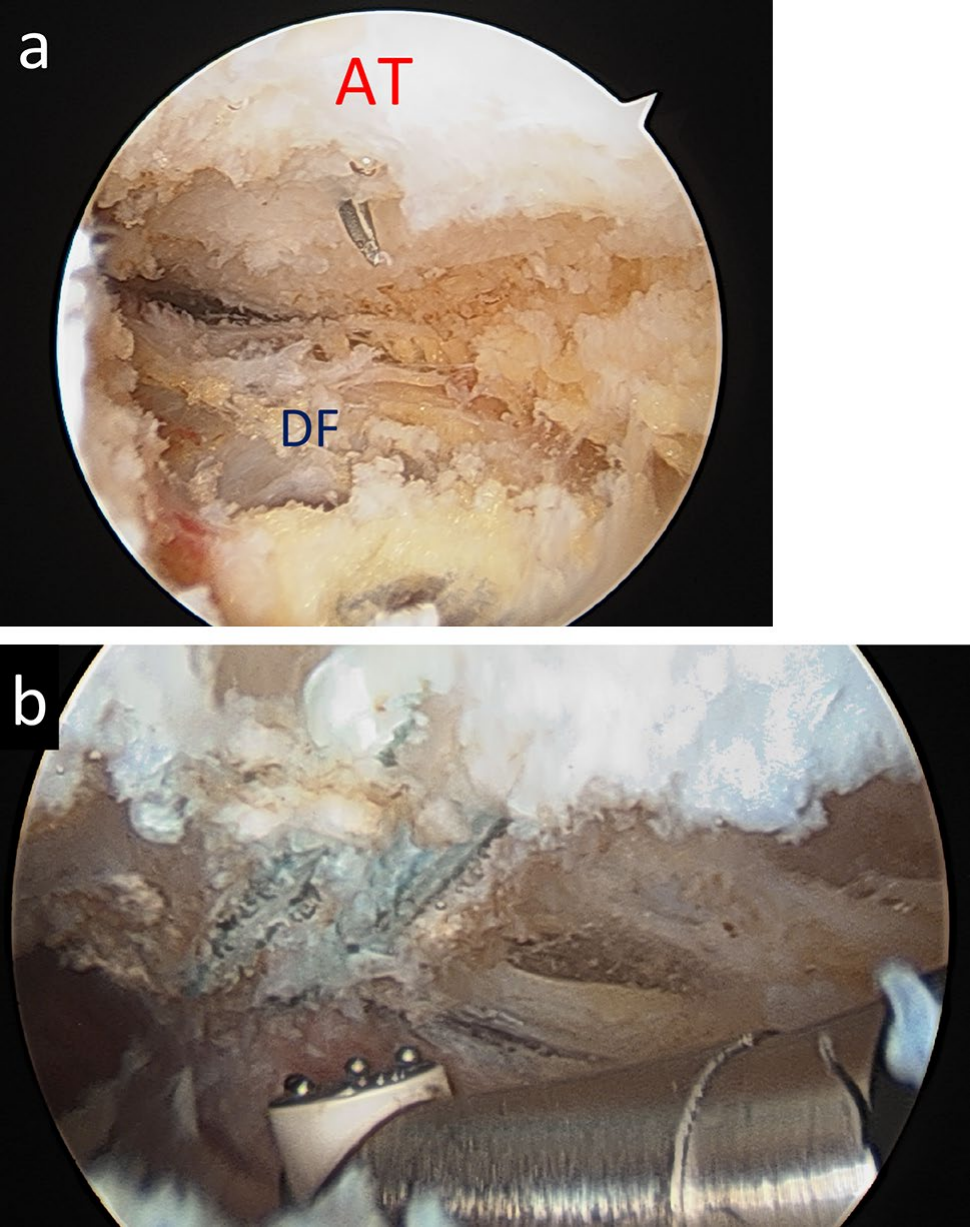
**a**, **b** Endoscopic appearances of the anterior (deep) aspect of the Achilles tendon, looking proximally. In both figures the Achilles tendon (AT) is at the top of the figure and the deep fascia (DF) covering the Flexor Hallucis Longus is at the bottom. In Fig. **b**, the knot of the Polyester suture is visible and more proximally the proximal end of the adhesions can be seen

Deep adhesiolysis was performed under general anaesthesia with patients in the recovery lateral position. Two percent chlorhexidine skin preparation was used together with a knee arthroscopy drape to optimise collection of irrigation fluid. Standard posteromedial and posterolateral portals [34] were used together with an Accessory Postero-Lateral portal [6]. A 30° 4-mm arthroscope was used together with a 4-mm Aggressive Plus shaver (Stryker, Amsterdam, The Netherlands) and 90° Radiofrequency Wand (90-S Cruise, Stryker, Amsterdam, The Netherlands) to move proximally along the anterior aspect of the tendon-releasing scar tissue. A hypodermic needle was passed through the tendon to aid orientation and determine proximal progress. Once adhesions were released, the endoscope could enter the pocket between the Soleus muscle and the fascia covering the Flexor Hallucis Longus muscle belly (Fig. 2). The adequacy of release can be determined by passively flexing and extending the ankle and first toe together and separately.

Following surgery, the endoscopic portals were closed with interrupted nylon sutures, simple dressing and the ankle received a compressive bandage for 24 h. No brace or splint was used and full weight-bearing and walking mobilisation was encouraged together with range of motion exercises. After 2 weeks, eccentric loading exercises were recommenced and running was permitted after 6 weeks. Patients were subsequently evaluated at 3 and 6 months following their additional surgery.

### Statistical analysis

Descriptive analysis was performed consisting of median (interquartile range) and mean (SD) for ATRS and mean (standard deviation) for HRHI. A direct comparison between those who were thought to have and thought not to have adhesions was not performed. Patients have been compared with historical cohorts in terms of ATRS and heel-rise height (Table 1).

**Table 1:** Historical cohorts of outcomes in terms of ATRS and HRHI for percutaneous and minimally invasive repair

	3 months	6 months		9 months		12 months	
	ATRS	ATRS	HRHI	ATRS	HRHI	ATRS	HRHI
Carmont et al. [7] Mean (SD)	42.5 (31)	73 (33)		83 (27)		89 (18)	
Median (IQR)	43 (17–93)	76 (26–100)		86 (31–100)		91 (48–100)	
Carmont et al. [8] Mean (SD)	50 (18)	76 (15.5)		86 (13.2)	66 (22)	90 (13)	82 (14)
Median (IQR)	45 (3–86)	77 (28–98)		89 (48–100)		93 (35–100)	
Carmont et al. [9] Mean (SD)	45 (20)	70 (16)	66 (26)	85 (10)	75 (21)	88 (13)	81 (22)
Median (IQR)	40 (13–82)	72 (39–97)		86 (10–100)		91 (54–100)	

*ATRS* Achilles tendon Total Rupture Score, *HRHI* Heel-Rise Height Index

*Means that patients were managed prior to routine Heel-Rise Height Index (HRHI) assessment

ATRS and Heel-Rise Height Index have been compared before and after surgical intervention, subsequent rehabilitation and return to activity using the Wilcoxon Signed-Rank test due to the small number of patients and the ordinal data of the ATRS.

## Results

Fourteen (5.6%) patients were identified as having adhesive complications, 4 patients had superficial adhesions and 10 had deep adhesions. The demographic details of these patients are shown in Table 2.

**Table 2:** Demographic details of patients with superficial and deep adhesions

Adhesion	Number	Age Mean (SD)	Gender Males:Females	Side Left:Right	Pre-injury Tegner Median (IQR)
Superficial	4	48.5 (3.4)	4:0	1:3	6.5 (3–9)
Deep	10	39 (6.6)	8:2	6:4	6.5 (5–9)

**Fig. 3:**
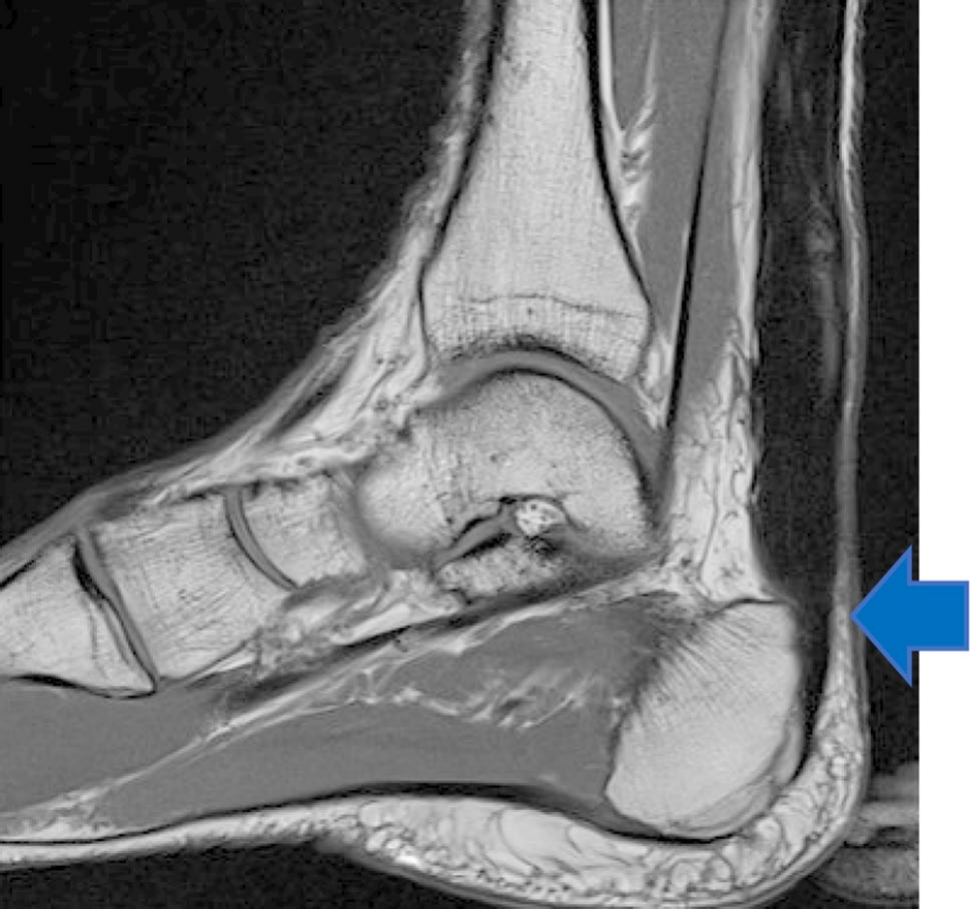
Pre-operative MRI T2 sagittal showing a very thickened but healed tendon, with some impingement on the postero-superior calcaneal tubercle (arrow)

At a mean (SD) of 10.5 (2.3) months following repair overall patient ATRS was median (IQR) of 65 (44.5–78) points and HRHI was at mean (SD) 81.5 (13.5)%. Nine patients, deemed to have deep adhesions, underwent pre-operative imaging consisting of MRI or ultrasonography at mean (SD) 15.5 (4.7) months following repair (Fig. 3). One patient did not receive imaging prior to endoscopic surgery. The antero-posterior (AP) diameter of the Achilles tendon of patients thought to have deep adhesions was at mean (SD) 15.6 (4.7) mm.

Following subcutaneous release, all four patients reported a reduction of stiffness and ATRS scores improved from being outliers to within the interquartile range (Table 1). Following endoscopic release of deep adhesions, all but one patient reported an improvement in symptoms and 9/10 patients demonstrated an improvement in HRHI, while ATRS scores improved in 7/10 patients (Table 3). Overall, ATRS improved in 13 out of 14 patients (93%) and HRHI in 11 out of 13 patients (85%).

**Table 3:** Achilles tendon Total Rupture Scores (ATRS) and Heel-Rise Height Index Scores (HRHI) over time following operative repair and subsequent surgical release (in bold)

Patient and adhesion	3 months	6 months		9 months		12 months		Tendon size/mm	Follow-up/months	ATRS	HRHI
	ATRS	ATRS	HRHI	ATRS	HRHI	ATRS	HRHI				
Superficial											
1	34	68	96	**90**	**96**	**98**	**96**		**12**	**98**	**96**
2	45	75	83	53	91	**80**	**96**		**12**	**80**	**96**
3*	17	34		**77**		**89**			**12**	**89**	
4	30	64	55	72	73	75	86		**14**	**90**	**91**
Median (IQR)	32.0 (20.3–42.3)	66.0 (41.5–73.3)		65 (28.3–86.8)		84.5 (76.3–95.8)				**85 (77.8–96)**	
Mean (SD)	31.5 (11.6)	60.3 (18.1)	78 (21)	60 (30.8)	86.7 (12.1)	85.5 (10.1)	96 (0.0)				94.3 (2.9)
Deep											
1	8	60	41	60	61	43	62	17 MRI	**16**	**90**	**86**
2	41	21	81	22	80	**54**	**80**		**16**	**56**	**83**
3	41	39	0	80	0	79	87	14 MRI	**18**	**58**	**86**
4	45	68	69	74	50	73	78	9.5 US	**18**	**91**	**88**
5	27	25	55	46	65	46	75	25 US	**24**	**72**	**83**
6	29	86	50	83	68	**82**	**62**	18 MRI	**14**	**96**	**80**
7	6	43	0	23	67	44	83	13 US	**17**	**34**	**75**
8	50	65	100	87	90	95	100	18 MRI	**14**	**92**	**100**
9	15	11	0	58	75	93	83	10.5 US	**16**	**95**	**92**
10	24	57	58	75	65	62	55	15 MRI	**19**	**67**	**65**
Median (IQR)	28 (13.3–42)	50 (24–65.8)		67 (40.3–80.8)		67.5 (45.5–84.8)				81 (57.5–92.8)	
Mean	28.6 (15.6)	47.5 (23.8)	64.9 (20.2)	60.8 (23.8)	69 (11.5)	67.1 (20.0)	76.5 (13.5)	15.6 (4.7)	**15.9 (3.3)**	**78.3 (18.9)**	**86.2 (9.5)**

Values in bold indicates values following surgical release. Values is bold relate to the patient’s scores and evaluation following surgical release of adhesions

*ATRS* Achilles tendon Total Rupture Score, *Sup* superficial

*Means that patient was managed prior to routine Heel-Rise Height Index (HRHI) assessment

**Fig. 4:**
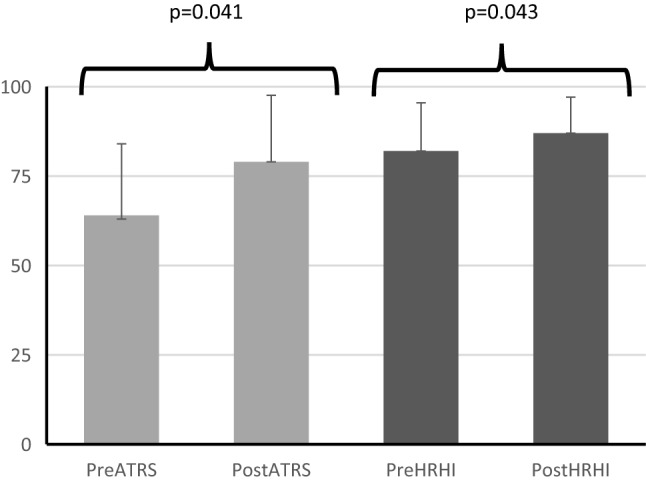
Pre- and post-ATRS and HRHI values for patients receiving superficial or deep adhesiolysis. All 14 patients reported ATRS and 13 patients had HRHI evaluation

At a mean (SD) of 15.9 (3.3)-month follow-up from initial rupture and repair, the patients reported ATRS scores of at a median (IQR) 85 (64.8–92.8) points, Tegner level 5 (3–9) and a mean (SD) HRHI 86.2 (9.5)%. Patients significantly improved both ATRS and HRHI following surgical release by at median (IQR) 16.5 (− 1.8–29.3) points (95% CI 1.61–28.2), (*p* = 0.041) and 5.6 (8.3)% (95% CI 0.07–11.2), (*p* = 0.043) (Table 3; Fig. 4).

There were no complications or recurrence of adhesions following the release of the subcutaneous adhesions. Following endoscopic adhesiolysis, there were no portal-related complications, iatrogenic sural nerve injury, deep venous thrombosis, tendon detachment or re-rupture. Some patients did report swelling around the tendon for 1–2 months following release.

## Discussion

The most important finding of this study is that on an individual patient level, superficial and deep adhesions significantly influenced clinical outcome following operative repair of the Achilles tendon. Adhesiolysis led to an improvement in outcome and function in over 85% of the patients.

Metz et al. [22] studied the effect of complications on ATRS in 211 survey respondents following Achilles tendon repair. Adhesions were grouped into minor wound complications, with a rate of 6%, where patients had a significantly lower score of 80 points compared with 89 points for those without these complications. The minimally important clinical difference has been suggested to be 10 points [3] while a minimal detectable change is 6.8 points [5]. In the present study, patients were prospectively observed reporting symptoms over time. Patients with superficial adhesions reported superficial tightness over the scar of the Achilles repair and identified the feeling of tightness localised to the area of skin tethering. Two of these adhesions were related to the sites of stab incisions for needle and suture passage in this repair technique. Subcutaneous adhesions usually became apparent during clinical follow-up as superficial tightness, the patient localising to skin tethering. This was usually appreciated by the 6 months’ time point with the restoration of ankle range of movement [25] and commencement of heel-rise exercises. This suggests that superficial adhesions can be recognised early following Achilles tendon repair.

Deep adhesions usually became apparent at the 9 months’ time point with patients complaining of stiffness to the tendon, a deep tightness to the calf and a band like medial discomfort passing from the back of the ankle to the medial malleolus, usually demonstrated with the palm of the hand. The differential diagnosis could include deep venous thrombosis, neurogenic leg pain and chronic regional pain syndrome. It is notable that two patients reported sciatica predating Achilles tendon rupture and had dysesthesia at the lower calf level.

It is difficult to predict which patients are likely to suffer from deep adhesions as multiple aetiological factors are likely. Notably, however, many patients went through a period of tendon pain and swelling at the 3–6-month period, associated with enthusiastic, possibly aggressive calf strengthening exercises. Zellers et al. [39] have noted that greater tendon cross-sectional area at 3 months was correlated with improved outcome at 12 months suggesting that having a thicker tendon earlier in recovery might lead to better outcome. Tendon thickness, however, increases up to 6 months following rupture and then decreases with remodelling [33]. In this series, tendons in those considered to have adhesions were thicker with diameter 15.6 (4.7) (9.5–25.0) mm on imaging performed at 15.5 (4.7) months following injury, compared with an AP diameter was 13.8 (12.1–15.6) mm at 4.6 (3.1–6.6) months in Zellers et al.’s series [38].

It is possible that the remodelling of tendons around the suture material may influence tendon thickness, biomechanical properties and the occurrence of adhesions. Tendons repaired with non-absorbable sutures during the period of increased rehabilitation may have led to tendon hypertrophy with surrounding inflammation potentially led to deep adhesion formation. On the contrary, this suture may have prevented excessive elongation of the tendon improving eventual outcome. In this series, all but one of the patients with deep adhesions were repaired using a non-absorbable suture. However, these patients were all repaired more recently when there was increased awareness of deep adhesion as a complication. A randomised prospective study is required to determine if the use of non-absorbable sutures is a relevant aetiological factor.

The surgical technique used might have a role in adhesion formation as due to concerns about body weight, distal suture pull-out and re-rupture, two patients with deep adhesions, had distal sutures passed through a transcalcaneal tunnel. These patients received a similar surgical technique according to Biljsma and van der Werken [4], however, a polyester-braided suture rather than an absorbable monofilament suture was used. The presence of the distal transosseous fixation with the polyester suture may have resulted in a less compliant tendon over the “zone” of repair and led to further deep adhesions and retrocalcaneal bursitis. The small number of patients treated using the transosseous technique makes comparison unreliable.

Simple preventive measures against superficial adhesions following Achilles tendon repair include soft tissue massage and early ankle range of movement exercises [10], although studies comparing early range of motion (ROM) versus cast immobilisation following repair [16, 17] did not comment on the incidence of adhesions. Surgical preventive techniques including an optimal closure of the fascia cruris and paratenon have been shown to be important following repair [26] and potentially subcutaneous layer closure. Percutaneous techniques such as those described by Amlang et al. [2] and Delponte et al. [10] use sutures which bypass the rupture site with knots tied through an incision proximal to the rupture site. Joannas et al. [15], Keller et al. [18] and Manegold et al. [20] report large case series (*n* ≥ 90) report only 4%, 2% and 1% complication rates mainly reruptures and knot prominence with no sural nerve lesions or infections.

It is also challenging to determine which patients may benefit from adhesiolysis and when it should be performed. Ahn et al. [1] recommend early open adhesiolysis in a case report where adhesiolysis was performed at 3 months following operative repair. The use of the ATRS and in particular, the responses to question related to stiffness may not be adequate to differentiate the presence of absence of adhesions and further research may be required in this area before definite recommendations can be made. Given that the healing tendon demonstrates increased metabolic activity for at least a year following repair [11], it is possible that early adhesion release may improve outcome by the 12 months following rupture. It may be that early surgery removes adhesions permitting improved motion with greater remodelling and tendon elasticity. Conversely, the removal of anterior blood vessels might impair subsequent healing and remodelling. Adhesiolysis after 12 months may be more beneficial as tendon healing has occurred and functional rehabilitation occurs with continued improvement from 1 to 2 years following rupture [31].

A limitation of this research is the small number of patients that were recognised as having adhesions. Together with the patients reported in the literature, this is a good indicator that adhesions are an infrequent complication or not observed or reported. The outcome variables studied tended to be close to a standard deviation below the mean scores of patient cohorts repaired using this technique prior to adhesion release. The validity of the clinical findings in this paper and whether they are true adhesions or scar tissue deserves consideration. The use of patient’s descriptions during interview/clinical evaluation means that there is a qualitative element to this study. It should be noted, however, operative release led to a significant clinical improvement from the patient’s perspective and an improvement in functional performance suggesting that adhesions is an appropriate term. Conversely the improvement may, however, have been due to the passage of time and further rehabilitation following tendon repair. The fact that the ATRS improved in only 7/10 patients following the release of deep adhesions might indicate that the questions in ATRS do not capture the consequences of adhesions. The method of data collection meant that it was not possible to identify if question 3 on the ATRS, asking about the limitation from tendon stiffness, was specific enough to appreciate the problem. In several patients, the overall score was low and limitation was reported in many questions. It is also notable that neuralgic features were present in those that did not improve, raising the possibility of co-morbidities influencing the score in addition to tendon problems.

The appreciation of the diagnosis and the clinical significance of adhesions has developed over 10 years of specialist evaluation of Achilles tendon surgery. Affected patients may have been reviewed earlier in the series and were not appreciated as having this complication. Similarly, different suture materials and slight modifications of surgical technique were performed over the time of this observation making the overall cohort heterogeneous, and thereby making more detailed analysis challenging. At this stage, the diagnostic features of deep adhesions on imaging are not fully appreciated and future investigation should involve both ultrasonography and MRI.

The implications of this research are that the outcome of patients with adhesions occurring during the management of patients following Achilles tendon rupture can be improved with adhesiolysis. Given adhesive complications may be more commonly found following surgical management, adhesiolysis will improve outcome of the surgically treated group compared with the non-surgical group in previous and future randomised controlled trials. Moreover, the complication of adhesions should be discussed during the consent process when patients choose between operative and non-operative treatment for the management of their Achilles tendon rupture.

## Conclusion

Adhesions were found to be an infrequent complication following minimally invasive repair of Achilles tendon rupture. Superficial adhesions were found to be associated with lower outcome scores. Symptoms of anterior tendon tightness and stiffness, thought to be due to deep adhesions were associated with a lower score in most patients. Surgical release of adhesions led to a significant improvement in outcome.
